# Avulsion Fracture of the Anterosuperior Iliac Spine: A Case Report

**DOI:** 10.7759/cureus.40374

**Published:** 2023-06-13

**Authors:** Hicham Azam, Bouchama Adnane, Larbi Benradi, Mohamed Belahcen

**Affiliations:** 1 Faculty of Medicine, Mohammed First University, Oujda, MAR; 2 Department of Pediatric Traumatology and Orthopedic Surgery, Mohammed VI University Hospital, Oujda, MAR

**Keywords:** spine, managment, iliac, fracture, anterosuperior

## Abstract

Avulsion fracture of the anterosuperior iliac spine is a rare injury, typically occurring in adolescents during sports activities. We present a case of a 13-year-old adolescent who experienced recent pelvic trauma, resulting in inguinal pain and functional impairment of the left lower limb. Clinical examination revealed pain upon hip mobilization and extension of the left hip joint. Pelvic X-ray revealed a fracture-avulsion of the anterosuperior iliac spine. The patient was managed conservatively with rest and unloading of the injured lower limb. Follow-up showed resolution of pain within a few weeks and the resumption of sports activities at six months.

## Introduction

Avulsion fractures of the pelvic apophyses are uncommon injuries. They are often misdiagnosed and mistaken for tendinopathies or muscle tears. These fractures typically occur in adolescents, between the onset of apophyseal ossification and their fusion to the corresponding pelvic tuberosities, especially in individuals engaged in intense sports activities.

These injuries occur at the cartilaginous growth plate of the apophysis. During this period, the apophyses, which serve as insertion sites for powerful muscles, represent a weak area in the musculoskeletal system of young individuals due to the high biomechanical stresses exerted by the muscles. The fractures usually result from a traction mechanism. Several cases of avulsion fractures of the anterior iliac spine have been described in the literature. We report a case of an avulsion fracture of the anterosuperior iliac spine (ASIS) in a young adolescent.

## Case presentation

A 13-year-old young adolescent presented to the emergency department with right inguinal pain and functional impairment of the lower limb, which occurred during sports activities. The patient had no significant medical history. Upon inspection, there were no obvious deformities, ecchymosis, or hematomas. Palpation revealed tenderness over the left ASIS (anterior superior iliac spine) and the underlying region. Resistance testing of hip flexion also elicited pain. There were no limitations in the range of motion, but an extension was moderately painful. The rest of the physical examination was unremarkable.

**Figure 1:**
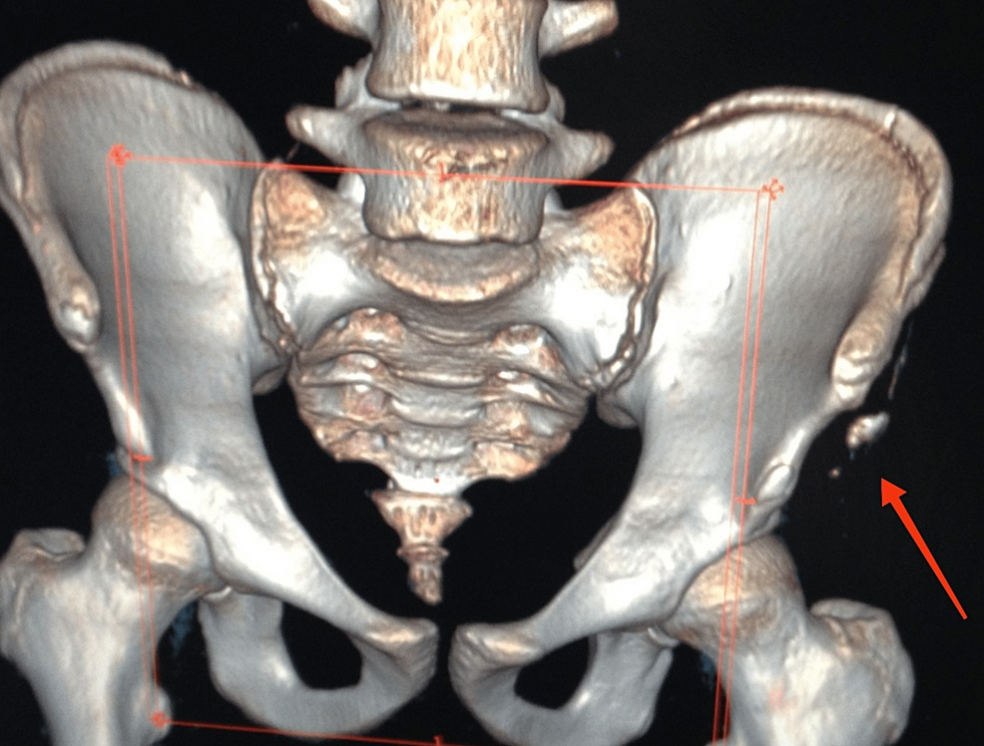
CT scan of the pelvis with 3D reconstruction 3D: Three-dimensional The red arrow is showing a fracture of the anterosuperior iliac spine.

Standard pelvic radiography revealed an avulsion of the ASIS. A computed tomography (CT) scan of the pelvis confirmed the findings of the radiograph (Figure 1).

The patient received orthopedic treatment consisting of rest, nonsteroidal anti-inflammatory drugs (NSAIDs), analgesics, and partial weight-bearing on the left lower limb for a duration of six weeks. No specific rehabilitation was advised. The patient experienced good functional outcomes, with pain resolution after a few weeks and a return to sports activities after six months. No complications were reported.

## Discussion

Avulsion fractures of the anterior iliac spines in athletic children and adolescents are rare acute injuries. They involve traumatic apophyseal avulsions, primarily affecting the anterior superior iliac spine (ASIS), but occasionally also the anterior inferior iliac spine [1,2]. These avulsion injuries occur in athletic children and adolescents, particularly during intense and forceful activities such as sprinting, jumping, or kicking. Avulsion of the ASIS occurs as a result of a sudden contraction or stretching of the sartorius or tensor fasciae latae muscles during rapid extension or resisted flexion of the thigh. A characteristic clinical sign is the elicitation of pain upon passive stretching of these muscles and resisted contractions during isometric tests. The sensitivity of the anterolateral thigh should always be checked to rule out the involvement of the lateral cutaneous nerve of the thigh [3].

The main differential diagnosis is a musculotendinous injury of the sartorius or tensor fasciae latae muscles. Age, careful palpation, and radiological assessment help differentiate between them. Complications are rare and can include pseudarthrosis or hypertrophic callus formation [4]. The clinical diagnosis of these apophyseal avulsion fractures is straightforward. The prominent symptoms are characterized by sharp, mechanical, sudden, significant, or even syncopal pain localized to the avulsion site, sometimes accompanied by clear functional impairment and limping, resembling a "muscle tear" presentation. The clinical examination reveals moderate pain upon passive mobilization of the hip [5]. Standard pelvic radiography, sometimes with oblique views, is usually sufficient to confirm the diagnosis by demonstrating the fracture and any displacement that may be present [6]. Treatment is typically conservative and based on bed rest, partial weight-bearing, and effective analgesic management [3,7,8].

Surgical reduction and internal fixation are rarely indicated, usually limited to cases with large fragment sizes and significant displacement [6]. Complications of conservative treatment primarily include exostoses and pseudarthrosis, while meralgia paresthetica represents the main complication of surgical treatment [9].

Early diagnosis of apophyseal injuries is crucial to promptly initiate appropriate treatment, including sufficient immobilization [10-12]. Additionally, when an apophyseal injury is diagnosed in an adolescent, other apophyses subjected to high tension should also be systematically evaluated [9]. Furthermore, better control of a child's physical activity by parents is essential in a society where sports have become a means of social advancement and where athletic performance can lead to physical overload in children, increasing the risk of apophyseal injuries [3,11].

## Conclusions

Apophyseal avulsion injuries are a specific pathology in athletic children and adolescents. Recognizing these injuries is crucial to avoid unnecessary and costly additional investigations. The patient's history plays a significant role in the diagnosis. An injury occurring in the context of sports activities, without direct impact, and with a history of previous pain suggestive of apophysitis, raises suspicion. Early and appropriate diagnosis and treatment of growth plate injuries are essential in preventing the occurrence of these types of fractures. Conservative treatment remains the gold standard and allows for a return to sports activities without sequelae.
